# Association of polygenic risk scores with low-density lipoprotein cholesterol levels and control: findings from the Hispanic Community Health Study/Study of Latinos (HCHS/SOL)

**DOI:** 10.64898/2026.09.16.26363269

**Published:** 2026-09-20

**Authors:** Christina G Hutten, Tamar Sofer, Brian W Spitzer, Molly Scannell Bryan, Jiehuan Sun, Victoria Persky, Martha L. Daviglus, Maria Argos

## Abstract

Background: Hypercholesterolemia is a prevalent cardiovascular (CVD) risk factor among Hispanics/Latinos; however, rates of cholesterol awareness and treatment are low in this population. Using a polygenic risk score (PRS) associated with low-density lipoprotein cholesterol (LDL) levels may help identify those who would benefit most from statin therapy. Previous LDL-PRS have been developed but they have not been properly evaluated for use in a highly diverse Hispanic/Latino population. Methods: The Hispanic Community Health Study/Study of Latinos (HCHS/SOL) is a prospective study that enrolled 16,415 Hispanic/Latino adults aged 18-74 years from four U.S. communities in 2008-2011. We assessed the association of twenty PRS with baseline LDL levels and LDL control among 11,669 HCHS/SOL participants with complete data and consent to conduct genetic research. Weighted PRS were calculated using effect estimates obtained from the PGS Catalog. Multivariable linear regression analysis was used to derive effect estimates (betas (&beta), 95% confidence intervals (CI)) for the association between each PRS, modeled continuously and by quintiles, with baseline LDL levels. Multivariable logistic regression was used to derive odds ratios and 95% CI for the association between each PRS and LDL control in statin users. Models were adjusted for predefined confounders and accounted for survey weights. Results: A PRS developed with PolyFun-pred, using European GWAS summary statistics, provided the largest incremental improvement for predicting LDL levels for the full sample (&Delta R2 0.114), Caribbean background (&Delta R2 0.096), majority African ancestry (&Delta R2 0.098), and majority Amerindian ancestry subgroups (&Delta R2 0.123). PRSCSx-EUR, using multiple ancestry GWAS and weights, improved PRS performance most for the Mainland background (&Delta R2 0.090) and majority European ancestry (&Delta R2 0.061) subgroups. For every one standard deviation increase across the 20 PRS asssesed, LDL increase varied between 3 and 14mg/dL. Among 1,323 statin users, the odds of LDL control were greatly reduced with increasing PRS but differed between PRS and Hispanic/Latino background groups and genetic ancestry groups. Conclusion: A polygenic risk score prioritizing functional annotations demonstrated superior performance for predicting LDL levels and LDL control across Hispanic/Latino subgroups. These results emphasize that further development of PRS is needed for improved risk prediction before clinical application in diverse populations.

## Full Text Availability

The license terms selected by the author(s) for this preprint version do not permit archiving in PMC. The full text is available from the preprint server.

